# A novel mutation in *GLUD1* causing hyperinsulinism-hyperammonemia in a patient with high density of homozygosity on microarray: a case report

**DOI:** 10.1186/s13256-016-0811-0

**Published:** 2016-02-02

**Authors:** John Odom, Maria Gieron-Korthals, Dorothy Shulman, Patricia Newkirk, Eloise Prijoles, Amarilis Sanchez-Valle

**Affiliations:** 1 USF Morsani College of Medicine, 12901 Bruce B Downs Blvd, Tampa, FL USA; 2 Department of Pediatrics, USF Health South Tampa Center for Advanced Healthcare, 2 Tampa General Circle, Tampa, FL 33606 USA; 3 USF Department of Neurology, 12901 Bruce B Downs Blvd, MDC 55, Tampa, FL 33612 USA; 4 Greenwood Genetic Center, 101 Gregor Mendel Circle, Greenwood, SC 29646 USA

**Keywords:** Developmental delay, *GLUD1*, Hyperammonemia, Hyperinsulinism, Hypoglycemia

## Abstract

**Background:**

Hyperinsulinism-hyperammonemia syndrome is the second most common cause of congenital hyperinsulinism and is easily treated with diazoxide; however, the symptoms in our patient were very difficult to control with typical medical therapy. To the best of our knowledge, neither our patient’s mutation, nor a case of hyperinsulinism-hyperammonemia presenting with dysmorphic features and intrauterine growth restriction has previously been reported.

**Case presentation:**

We describe a 2-year-old Hispanic girl with an unusual presentation of dysmorphic features and intrauterine growth restriction who was later found to have hyperinsulinism-hyperammonemia syndrome. Chromosomal microarray analysis revealed no copy number variants but demonstrated a high density of noncontiguous regions of homozygosity consistent with limited outbreeding. Sequencing of her *GLUD1* gene revealed a previously undescribed mutation of cytosine to thymine at position 1519 resulting in an amino acid change of histidine to tyrosine at position 507. Although no functional studies were performed, function prediction tools in combination with our patient’s phenotype support the hypothesis that the mutation is deleterious. Despite treatment with a maximum dose of diazoxide (15 mg/kg/day), phenobarbital (8.5 mg/kg/day divided twice daily) and a protein-restricted diet, she has global developmental delay, and continues to have seizures and recurrent episodes of hypoglycemia.

**Conclusions:**

It remains unclear if her clinical presentation can be solely explained by hyperinsulinism-hyperammonemia syndrome or is the result of an undiagnosed recessive disorder related to her homozygosity. It is our hope that clinicians may learn from our patient when formulating treatment plans for refractory cases of hyperinsulinism-hyperammonemia and avoid the morbidities associated with delayed diagnosis and treatment.

## Background

Hyperinsulinism-hyperammonemia syndrome (HI/HA; OMIM 606762) was first described in 1996 by Zammarchi *et al*. and since then has been shown to be the result of a gain of function mutation in the *glutamate dehydrogenase 1* (*GLUD1*) gene [1, 2]. It is currently the second leading cause of hyperinsulinism in hypoglycemic babies. This mutation leads not only to the aberrant secretion of insulin from the pancreas, but also impaired ammonia processing by the liver [3]. Patients with HI/HA are usually born at term with normal birth weights, and they typically present with signs and symptoms of hypoglycemia after they are several months old; for some, the hypoglycemia may be mild and present at the time of weaning [3, 4]. Patients with HI/HA typically present with recurrent episodes of hypoglycemia and hyperammonemia. At the time of hypoglycemia, these patients have inappropriately elevated serum insulin levels, low fatty acid and low ketone body levels [5]. They are typically asymptomatic for the hyperammonemia, but are symptomatic for hypoglycemia, with 43 % of patients developing epilepsy [4]. Their hypoglycemia will typically occur after fasting or after the ingestion of a protein meal. Patients also show an exaggerated response to intravenous glucagon [2]. HI/HA can result in permanent brain damage if left untreated; however, risk of brain damage seems to be due to delays in diagnosis rather than the nature of the underlying genetic defect [6]. Thus, it is important to consider HI/HA as a diagnosis in all babies with hyperinsulinemia-related hypoglycemia. Serum ammonia levels should be measured; however, some patients with HI/HA can have ammonia levels within the normal range. A leucine tolerance test can be used to rule in HI/HA, as patients with HI/HA will develop leucine-induced hypoglycemia. A definitive diagnosis is obtained by direct sequencing of the *GLUD1* gene [5]. It is our hope that in sharing this child’s unusual presentation with the medical community, other children with similar presentations can have their care expedited, and undue mortality and morbidity can be avoided.

## Case presentation

**Fig. 1:**
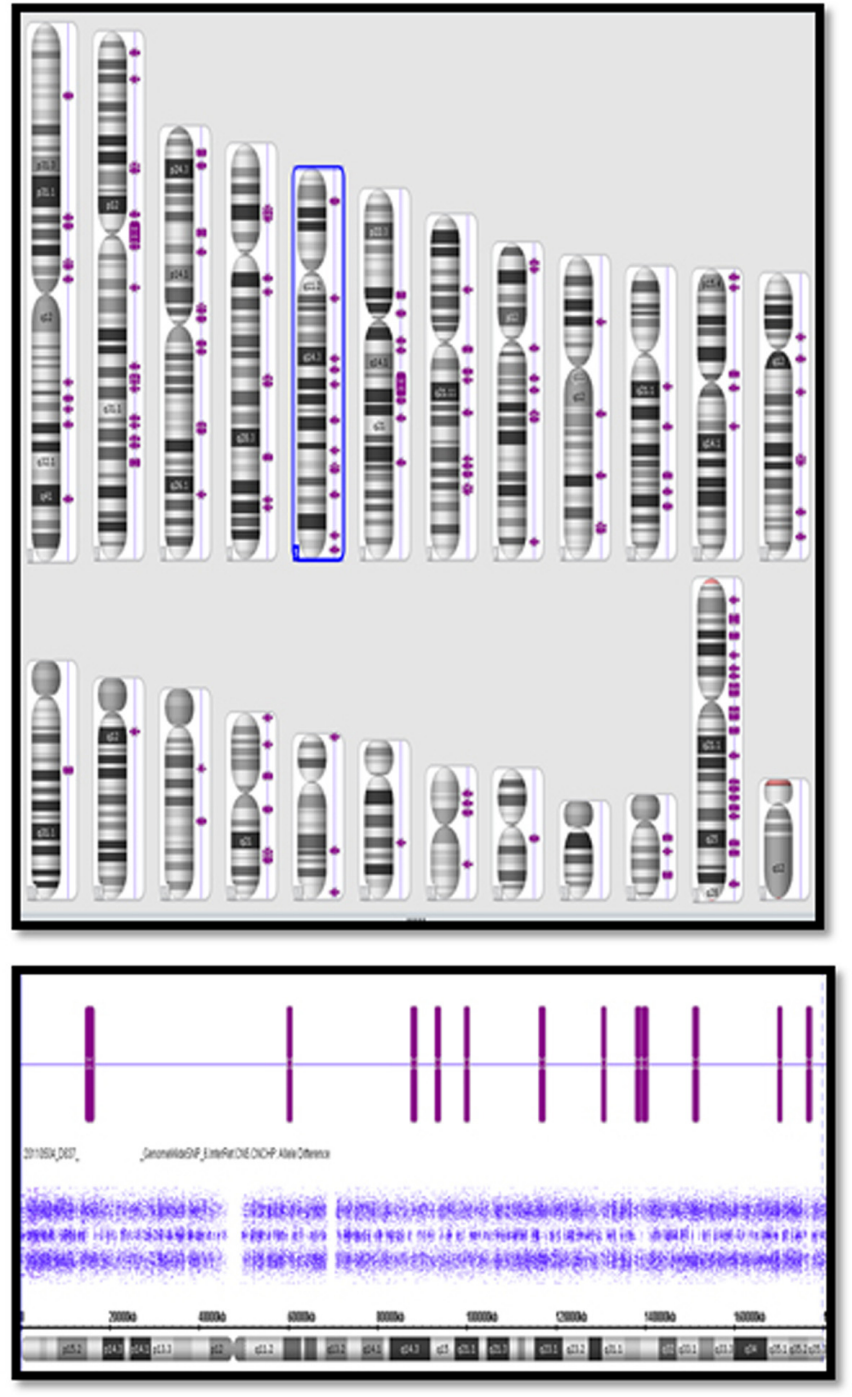
Chromosomal microarray analysis. Showing both the karyotype homozygous regions (>1 Mb) in the upper image and the specific single nucleotide polymorphism allele calls in the lower image. Purple bars indicate the absence of heterozygosity with the gaps in the midline heterozygote tract

We report the case of a 2-year-old Hispanic girl with HI/HA (OMIM 606762) who presented at 2 months of age with a history of intrauterine growth restriction (IUGR), patent foramen ovale, peripheral pulmonic stenosis and dysmorphic features. She was the product of an uncomplicated pregnancy and born at 39 weeks’ gestation by vaginal delivery with a birth weight of 1.9 kg (<first percentile) and a length of 42.18 cm (<first percentile). She had a wide anterior fontanel, widely spaced nipples, posteriorly rotated and low-set ears, poorly defined philtrum, blue sclera and double posterior hair whorls. A chromosomal microarray analysis (Affymetrix 6.0) revealed a normal female with a high density of noncontiguous regions of homozygosity. She had multiple short runs (1 to 10 Mb) of allele homozygosity throughout the genome, consistent with limited outbreeding (Fig. 1). Her family history was unremarkable except that consanguinity was suspected as both parents were from a small village in Mexico.

At 8 months of age, she was admitted to our hospital with new onset seizures. Her seizures were described as episodes of unresponsiveness and staring. She had associated cyanosis with most episodes and body stiffness occurred with some episodes. An electroencephalogram showed poorly organized and slower than expected for age cerebral activities, and epileptic discharges in her left centroparietal region. The findings were consistent with encephalopathy and indicated predisposition for partial onset seizures. A non-contrast brain magnetic resonance imaging (MRI) showed no abnormalities or pathology.

She was found to be hypoglycemic (39 mg/dL). Her hypoglycemia workup revealed an insulin level of 6 μU/mL (relatively elevated for a blood glucose of 44 mg/dL), negative urine ketones, low beta-hydroxybutyrate, and hyperammonemia (150 to 200 μmol/L). She was managed with dextrose and required a glucose infusion rate >8 mg/kg/minute. She was given a trial of Buphenyl (sodium phenylbutyrate), but her ammonia did not normalize and she remained asymptomatic for hyperammonemia. HI/HA was suspected and sequencing of her *GLUD1* was ordered. Initially, her hypoglycemia responded well to 10 mg/kg/day of diazoxide, and her seizures were controlled with levetiracetam. She was discharged home on a protein-restricted diet, and she was referred to the state’s Early Steps program for evaluation of developmental delay. Results from the *GLUD1* sequencing showed a C to T mutation at position 1519 resulting in an amino acid change of histidine to tyrosine at position 507. This mutation has not been described previously in the literature; however, it was predicted to be deleterious by SIFT and PolyPhen-2.1.0 programs. Parental DNA testing confirmed the mutation to be *de novo*, supporting the diagnosis of this autosomal dominant metabolic disorder. Functional studies of the gene were not performed as we thought that the patient’s phenotype (HI/HA) would only be possible if said mutation was in fact deleterious.

At 18-months old, she presented to our emergency department for seizures and hypoglycemia (19 mg/dL). In our hospital, she did not respond to diazoxide and required a high infusion rate of dextrose administered intravenously to maintain a normoglycemic state. She responded to an increase in diazoxide to 15 mg/kg/day, given as a fractional dose every 8 hours. She continued to have hyperammonemia but was asymptomatic. She remained non-responsive to dietary management.

Global developmental delay was apparent. She sat at 8 months, started walking at 30 months, and at 30 months she vocalized but spoke no specific words other than “mom” and “dad”. She was also having behavioral problems: hitting and biting. She is currently on a regimen of a protein-restricted diet, diazoxide 15 mg/kg/day, and phenobarbital 8.5 mg/kg/day. She has had recurrent hypoglycemia, particularly during intercurrent illness. She was started on cornstarch, without improvement in her blood glucose levels. Noncompliance was ruled out by measuring her diazoxide level. Nifedipine 0.5 mg/kg/day divided three times daily was added with some improvement in her hypoglycemia. The dose has recently been increased to 0.8 mg/kg/day. Somatostatin analog therapy will be considered if higher doses of nifedipine are not tolerated and hypoglycemic episodes persist.

## Conclusions

HI/HA can result in permanent brain damage if left untreated. Thus, it is important to consider HI/HA in the diagnosis of babies with hyperinsulinemic-related hypoglycemia and treat it promptly. Early recognition and appropriate treatment of HI/HA are essential to avoid developmental delay and permanent neurologic damage [7, 8]. Studies have shown that the level of developmental delay associated with congenital hyperinsulinism is typically associated with the severity of the hypoglycemia and the promptness with which episodes are controlled. Our patient initially responded quickly to the medical management with diazoxide. However, after several months of treatment her hypoglycemia returned.

Despite early diagnosis and treatment of our patient’s HI/HA, she has significant global developmental delays. It remains unclear if her developmental delays are a result of complications from her HI/HA, such as chronic early hypoglycemia and chronic mild hyperammonemia, or are related to yet another condition. In 2001, de Lonlay *et al*. reported on the cases of 12 unrelated patients with HI/HA; only two patients had psychomotor retardation, both of whom presented in the first 3 days of life [7]. Bahi-Buisson *et al*. analyzed 22 patients for neurological outcome and they reported that 17 of 22 had learning disabilities; however, only two had pyramidal involvement and one had dystonia [9].

Neither a H507Y mutation, nor a case of HI/HA presenting with dysmorphic features and IUGR has previously been reported. SIFT and PolyPhen-2.1.0 programs predicted this mutation to be deleterious, resulting in a *GLUD1* with strongly reduced inhibition by GTP. The loss of inhibition by GTP is a common mechanism of disease progression in patients with HI/HA and is most likely the case for our patient; although it is important to note that no functional studies were performed on the gene.

Our patient initially responded well to diazoxide; however, more recently, she has required the addition of a calcium channel blocker to normalize her blood glucose. de Lonlay *et al*. reported that one of 12 of their patients did not respond to diazoxide. Although their patient presented at 7 months, similar to our patient, theirs did not have any psychomotor retardation [7].

HI/HA is not usually associated with severe developmental delay, IUGR or dysmorphic features. Presumably, our patient’s IUGR and dysmorphic features are not due to her HI/HA, but to an unknown recessive condition. It also remains unclear if her developmental delays are a result of complications from her HI/HA or are related to another condition. It is our hope that in the future, clinicians may learn from our patient when facing refractory cases of HI/HA and avoid the morbidities associated with late diagnosis and treatment.

## Consent

Written informed consent was obtained from the patient’s parents for publication of this case report and accompanying images. A copy of the written consent is available for review by the Editor-in-Chief of this journal.
